# Vascular and Glycemic Modulation by *Prosthechea livida*, an Orchid Used as Traditional Medicine

**DOI:** 10.3390/ph18060881

**Published:** 2025-06-12

**Authors:** Angélica Flores-Flores, Samuel Estrada-Soto, Marlen Miuler Mulero-Navarrete, Rogelio Hernández-Pando, Oswaldo Javier Enciso-Díaz, Luis Arias-Durán, Blanca Bazán-Perkins, Rafael Villalobos-Molina

**Affiliations:** 1 Laboratorio de Inmunofarmacología, Instituto Nacional de Enfermedades Respiratorias Ismael Cosío Villegas, Mexico City 14080, Mexico; perkins@unam.mx; 2 Escuela de Medicina y Ciencias de la Salud, Tecnológico de Monterrey, Monterrey 64700, Mexico; 3 Facultad de Farmacia, Universidad Autónoma del Estado de Morelos, Cuernavaca 62209, Mexico; marlen.mulero@uaem.edu.mx (M.M.M.-N.); adl_ff@uaem.mx (L.A.-D.); 4 Sección de Patología Experimental, Departamento de Patología, Instituto Nacional de Ciencias Médicas y Nutrición Salvador Zubirán, Mexico City 14080, Mexico; rhdezpando@hotmail.com; 5 Facultad de Ciencias Biológicas y Agropecuarias, Universidad Veracruzana, Córdoba 94500, Mexico; oenciso@uv.mx; 6 Unidad de Biomedicina, Facultad de Estudios Superiores-Iztacala, Universidad Nacional Autónoma de México, Tlalnepantla 54090, Mexico; villalobos@unam.mx

**Keywords:** diabetes, hypertension, orchid, *Prosthechea livida*, traditional medicine

## Abstract

**Background:** Mexico, one of the world’s most biodiverse countries, holds a rich tradition of using medicinal plants to manage chronic diseases such as type 2 diabetes and hypertension. Despite their historical significance, the scientific validation of these plants’ mechanisms and safety remains limited. Natural products have shown potential in improving insulin sensitivity, reducing insulin resistance, and promoting vasodilation. *Prostachea livida (Lindl.) W.E.Higgins*, a native orchid, is believed to possess therapeutic properties, yet its pharmacological effects are unexplored. **Objective**: The current investigation is aimed to bridge traditional knowledge and scientific evidence by investigating the antidiabetic, vasodilatory and antihypertensive activities of a 1:1 dichloromethane and methanol extract from *Prosthechea livida* bulbs, alongside an evaluation of its pharmacological safety. **Methods:** Antidiabetic effects of the extract were evaluated in a non-insulin-dependent mouse model using a 100 mg/kg dose. Vasodilatory activity was assessed ex vivo using rat aortic rings, exploring its mechanism through calcium channel blockade. Antihypertensive effects were evaluated in spontaneously hypertensive rats, while acute and subacute toxicity tests were conducted in a murine model. **Results**: The extract significantly reduced glycemia between 1, 3, 5, and 7 h compared to the positive control (* *p* = 0.04, *** *p* < 0.001) and induced vasorelaxation but showed no antihypertensive effects in vivo. Toxicity tests indicated no severe damage, though elevated transaminase activity and increased liver weight were observed. Histopathological analysis revealed minimal hepatocellular lesions with active regeneration. **Conclusions**: *Prosthechea livida* demonstrates potential in the discovery of active metabolites to treat diabetes, with significant hypoglycemic and vasorelaxant effects and promising pharmacological safety. Further research is needed to fully understand its therapeutic applications and ensure its safe integration.

## 1. Introduction

Natural products (NPs) are among the principal sources used by the Mexican population to treat various diseases, ranging from non-communicable to chronic degenerative diseases, such as hypertension and diabetes [1]. Diabetes is a serious, chronic condition that occurs when the body cannot produce enough insulin or cannot use the insulin that it does produce, leading to high levels of glucose [2]. Over time, this disease can lead to other complications, such as vascular trauma, retinopathy, heart disease, nephropathy, and diabetic neuropathy. There is scientific evidence of the relationship between vascular diseases and glycemic disorders. Hyperglycemia has been identified as a critical factor that elevates the risk of developing vascular complications in patients with diabetes [3]. This association is evident in individuals with type 2 diabetes (T2D), where inadequate glycemic control has been correlated with an increased incidence of both microvascular and macrovascular complications [4]. Approximately 589 million people are affected by diabetes, which can even be fatal [5]. Observational clinical studies have mentioned the importance of maintaining optimal glycemic levels as a key strategy to reduce the burden of vascular complications. Furthermore, these studies emphasize the need for the regular monitoring and effective management of additional cardiovascular risk factors, particularly blood pressure, in patients with T2D [6]. Natural products have been the subject of extensive research in the management of diabetes and hypertension. Several natural compounds and extracts have shown potential in improving blood glycemia and blood pressure by enhancing insulin sensitivity or by promoting vasodilation [7]. It is essential to understand that drug development involves extensive testing in preclinical studies and multiple phases of clinical trials to ensure the pharmacological safety and efficacy of the evaluated compound. While some approaches may show promising results in the early stages of research, not all of them will eventually become approved treatments for diabetes and/or cardiovascular diseases. Therefore, ongoing research and clinical trials are crucial in bringing new and more effective drugs or natural products to the market for diabetes and hypertension management [8]. In this research, we focused on a single species in the orchid family, as there have been a wide variety of studies on different orchid species exploring their pharmacological and phytochemical properties. There is a greater interest in these plants as ornamental flowers and in the perfume and cosmetics industry; however, there are limited studies on their pharmacological properties related to chronic diseases.

Mukherjee and Jagtap (2020) reported on up to 20 orchid species for diabetes management and other related disorders; parts of the whole plant, such as roots, leaves, pseudobulbs, tubers, and chewed plant material, were prepared in decoctions and infusions [9]. These orchids are used for diabetes, hyperglycemia, and urinary problems, with *Prostechea karwinskii (Mart.) J.M.H* being the most commonly used orchid in Mexico and Latin America. Additionally, pharmacological evidence across 17 species, supported by animal studies and clinical trials, suggests the antidiabetic potential of orchids and related benefits such as antihyperglycemic, antioxidant, and anti-insulin resistance, along with improvements in retinopathy and anti-obesity effects [9]. In Mexico, *Prosthechea karwinskii* is traditionally used by Indigenous communities to treat coughs, wounds, burns, and diabetes. Bioactive compounds from this plant have been shown to reduce obesity, insulin resistance, and cardiovascular risk in a model of metabolic syndrome [10,11]. On the other hand, studies indicated that extracts from *Prosthechea karwinskii* showed anti-inflammatory and antioxidant properties in a rat model, with roles played by LTB4 and nitric oxide in its gastroprotective effect [12]. Another species, *Prothechea michuacana W.E. Higgins (LaLlave & Lex)*, showed nephroprotective activity in a study on cisplatin-induced acute renal failure in rats [13].

**Figure 1:**
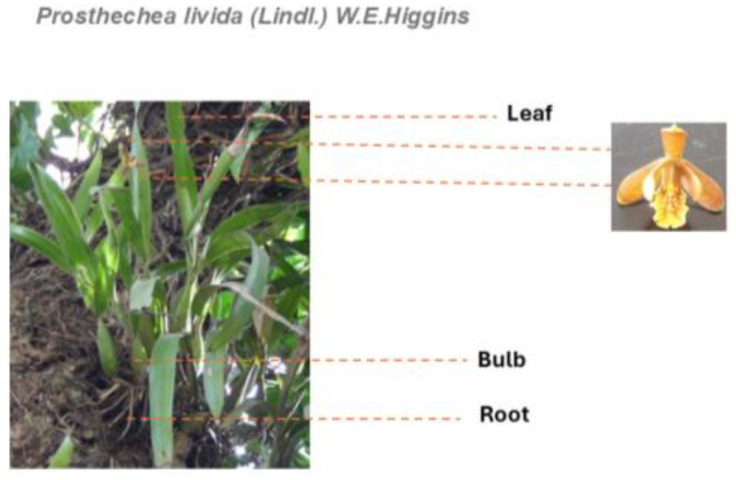
Photography of *Prosthechea livida (Lind) W.E. Higgins* captured by Oswaldo Javier Enciso- Díaz.

*Prostachea livida (Lindl.) W.E.Higgins* is an epiphytic orchid with an elliptical pseudobulb and one or two apical leaves (Figure 1). It blooms throughout the year, with an apical inflorescence measuring 4 to 11 cm and producing 3 to 7 flowers. This orchid is found in humid and seasonally dry forests, forming mats on trees, cacti, and rocks. It is distributed across a number of States of Mexico, such as Chiapas, Oaxaca, San Luis Potosí, Tabasco, Tamaulipas, and Veracruz [14].

Therefore, this study aimed to explore the pharmacological potential of *Prostachea livida (Lindl.) W.E. Higgins* in the treatment of diabetes and hypertension. Given the limited scientific evidence available on its efficacy and safety, this research focused on the generation of 1:1 dichloromethane and methanol extracts from its bulbs to evaluate their pharmacological effects as an adjuvant in lowering blood glucose levels, vasodilatory effects, and toxicological profiles, thus contributing to the validation of its traditional use by the Mexican population as a medicinal plant.

## 2. Results and Discussion

### 2.1. Oral Glucose or Sucrose Tolerance Tests

**Figure 2:**
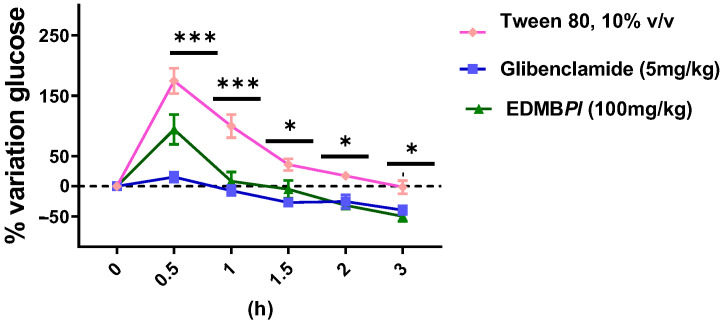
Effect of EDMB*Pl* on blood glucose after a single oral load of 2 g/kg of glucose in male normoglycemic mice *n* = 6 at 0.5, 1, 1.5, 2, and 3 h, compared to the vehicle. Statistically significant differences between the treatments were tested by two-way ANOVA, followed by a Tukey *t*-test. * *p* = 0.01,0.04, *** *p* < 0.001.

**Figure 3:**
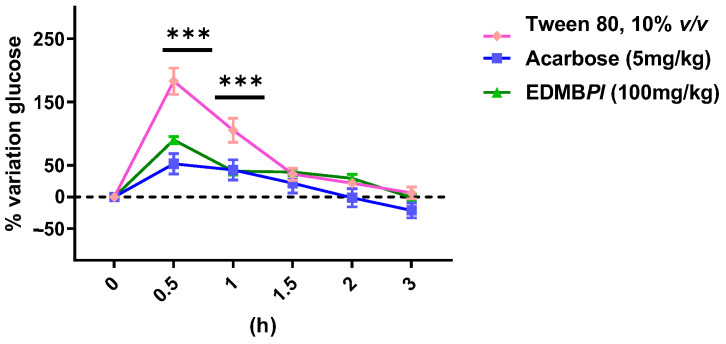
Effect of EDMB*Pl* on blood glucose after a single oral load of 2 g/kg of sucrose in male normoglycemic mice *n* = 6 at 0.5, 1, 1.5, 2, and 3 h, compared to the vehicle. Statistically significant differences between the treatments were tested by two-way ANOVA, followed by a Tukey-test. *** *p* < 0.001.

Hypertension and diabetes have a very close relationship. It is well established that type 2 diabetes can contribute to the development of cardiovascular complications such as hypertension, though the reverse is not true. However, the exact link between these two diseases remains unclear [15]. Treatments are available for both hypertensive and diabetic patients, but challenges arise when these conditions coexist or when type 2 diabetes triggers the onset of hypertension or chronic kidney disease [7]. In this context, *Prostachea livida* has been studied in animal models to assess its effects on diabetes and hypertension, as well as its pharmacological safety. It is important to note that, while there is no prior scientific evidence supporting its uses for these conditions, *Prostachea livida* is commonly used in traditional Mexican medicine to treat hypertension and other ailments. Furthermore, the genus *Prosthechea* has been the subject of two previous ethnopharmacological studies that have reported anti-inflammatory effects and bioactive compounds against components of metabolic syndrome [10,11]. In our study, the oral glucose tolerance test (OGTT) was performed; it is considered a gold-standard test for diagnosing diabetes to estimate glucose uptake according to the World Health Organization [5,16,17]. As shown in Figure 2, *Prosthechea livida bulb* extract (EDMB*Pl*) at a dose of 100 mg/kg reduces the percentage of glycemia variation between 30 min and 3 h, displaying an effect like that of the positive control (glibenclamide 5 mg/kg). In addition, the fact that the extract opposes the hyperglycemic peak indicates that the effect may be related to the inhibition of the Na^+^-dependent glucose transporters or the transporter GLUT2 [18]. Furthermore, the effect observed after 30 min may be attributed to secretagogue action and increased insulin sensitivity [19]; further experiments are necessary to explore those mechanisms of action. Subsequently, the oral sucrose tolerance test was conducted to evaluate the extract’s effect on disaccharide hydrolysis. Intestinal disaccharidases cleave disaccharides such as sucrose and maltose into monosaccharides. When sucrose is cleaved, glucose is released, which is absorbed by Na^+^-glucose co-transporters [20]. As shown in Figure 3, EDMB*Pl* reduces glycemia variation from 30 min to 3 h, comparable to the positive control group (acarbose 5 mg/kg), which inhibits α-glucosidase enzymes, which break down dimeric carbohydrates into monomers, such as glucose and fructose, that are absorbed into the bloodstream for energy. Their inhibition leads to a decrease in the absorption of carbohydrates in the gut. These results suggest that EDMB*Pl* is associated with the inhibition of glucose uptake from the intestinal lumen, likely due to its inhibitory effect on the intestinal α-glucosidase complex, which plays a key role in carbohydrate digestion [21,22].

### 2.2. Non-Insulin-Dependent Model (NIDD) of T2 Diabetes

**Figure 4:**
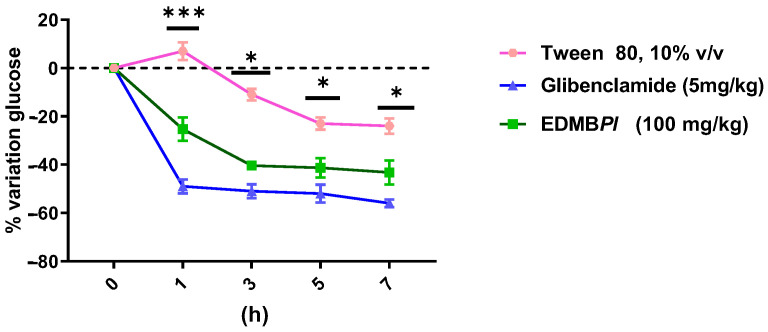
Evaluation of the antidiabetic activity of EDMB*Pl* on blood glucose in streptozotocin-nicotinamide-induced diabetic mice, where a glucose reduction is observed at 1, 3, 5, and 7 h compared to the positive control *n* = 6. Statistically significant differences between the treatments were tested by two-way ANOVA, followed by a Tukey test. * *p* = 0.04, *** *p* < 0.001.

After observing the antihyperglycemic effect of EDMB*Pl* in normoglycemic animals, we evaluated its acute antidiabetic effect in an STZ-induced diabetic mouse model. As shown in Figure 4, EDMB*Pl* at a dose of 100 mg/kg reduced glycemia in diabetic mice from the first few minutes up to seven hours of experimentation, exhibiting a similar effect to glibenclamide (5 mg/kg). This suggests that the extract may act similarly to sulfonylureas by stimulating insulin secretion in pancreatic β cells [23]. However, extrapancreatic mechanisms related to insulin sensitization cannot be ruled out [24].

### 2.3. Vasorelaxant Effect of EDMBPl

**Figure 5:**
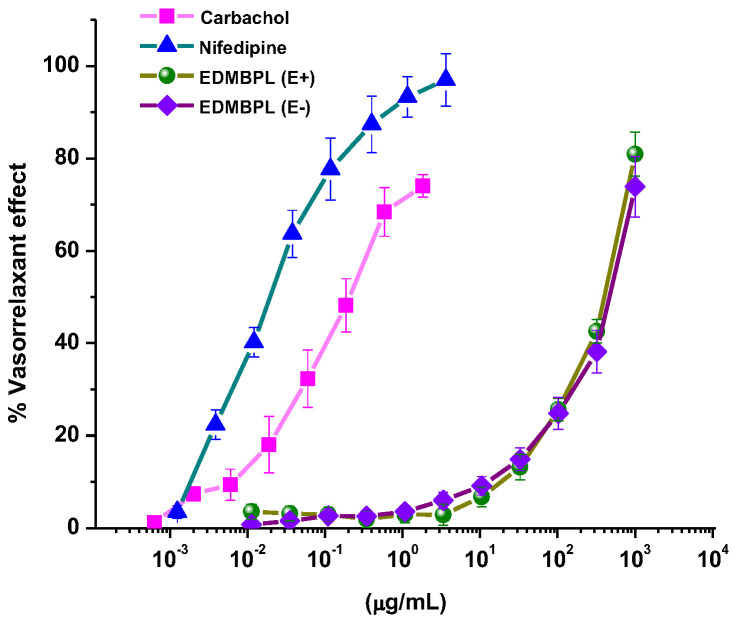
Vasorelaxant effect of EDMB*Pl* in the presence (E+) and absence of the endothelium (E−). No significant difference is seen in the percentage of relaxation in the presence and absence of the endothelium. Each point represents the mean ± S.E.M. for five animals.

In addition to our experiments on diabetes, we aimed to investigate whether EDMB*Pl* had a hypertensive effect due to its potential relationship with this disease. Therefore, EDMB*Pl* was tested on rat aortic rings to evaluate its vasorelaxant effect. As shown in Figure 5, EDMB*Pl* shows a concentration-dependent effect in the presence of endothelium, with a maximum effect (E_max_) of 80.9 ± 4.7% and effective concentration 50 (EC_50_) of 502.5 ± 2.5 µg/mL. In the absence of endothelium, the maximum effect was E_max_ = 73.9 ± 6.5% with an EC_50_ = 536 ± 4.6 µg/mL. The similar response observed in both conditions suggests that the extract may exert its vasorelaxant effect directly on the vascular smooth muscle [25].

### 2.4. Determination of the Mechanism of Action of EDMBPl

**Figure 6:**
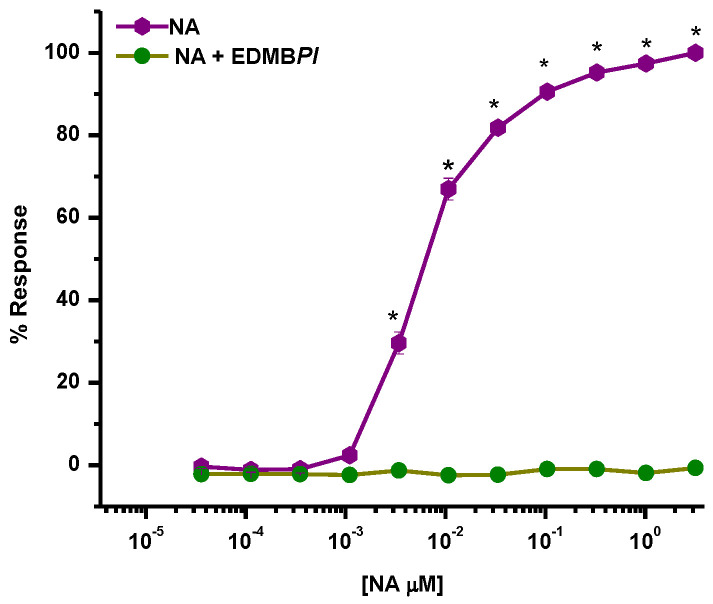
Effect of EDMB*Pl* on the contraction induced by NA (0.1 nM to 0.1 μM) in aortic rings without the endothelium, where a significant difference is seen in the percentage of responses induced by NA. Each point represents the mean ± S.E.M. for five animals. A statistical difference was considered when * *p* < 0.05.

We determined the possible mechanism of action of EDMB*Pl* when it was added to aortic rings lacking vascular endothelium and explored with functional experiments. First, we explored the participation of α-adrenergic receptors (Figure 6); a non-competitive antagonist effect (a complete blockade of NA-induced contraction) can be observed, suggesting a possible functional antagonism caused by a downstream interaction in post-agonist-receptor binding signaling [26].

An intracellular calcium increase plays a role in the activation of various disease processes, including hypertension. When calcium ions enter the cells and bind to calmodulin, this activates the enzyme myosin light chain kinase, which then phosphorylates myosin, allowing it to bind to actin and generate contractions [27,28].

**Figure 7:**
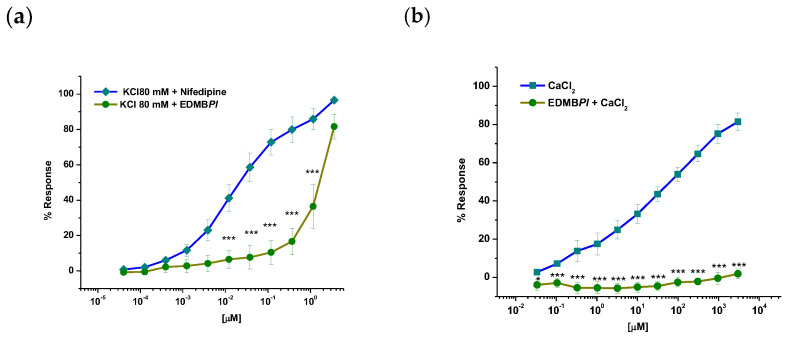
(**a**) Effect of EC_50_ EDMB*Pl* on the contraction-induced KCl (80 mM) in aortic rings without the endothelium, (**b**) effect of EC_50_ EDMB*Pl* on the contraction induced by cumulative concentrations of CaCl_2_, in Ca^2+^ free Krebs–Henseleit solution. Each point represents the mean ± S.E.M. for five animals. Statistically significant differences between the treatments were tested by two-way ANOVA, followed by a Bonferroni test. * *p* = 0.02, *** *p* < 0.001.

In Figure 7a, pre-incubation with the EC_50_ of EDMB*Pl* followed by a concentration–response curve (CRC) to CaCl_2_-induced contraction can be observed; the extract avoided contraction, suggesting an effect on the smooth muscle of blood vessels via calcium channel blockade [29]. This is further confirmed by the CRC for the administration of 80 mM KCl, which led to membrane hyperpolarization through BK_Ca,_ allowing calcium entry through Cav 1.2 calcium channels.

In this sense, EDMB*Pl* induced a concentration-dependent vasorelaxant effect (Figure 7b), corroborating its ability to block calcium channels and thereby reducing vasocontractions. As the smooth muscle begins to relax, a comparison with the control (nifedipine) shows that the EDMB*Pl* extract has greater efficacy (E_max_ = 81.1 ± 2.2 %); however, it is less potent than the positive control.

### 2.5. Antihypertensive Study

**Figure 8:**
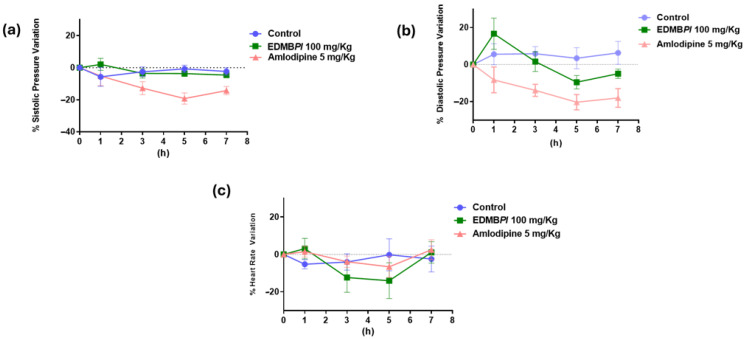
(**a**) Systolic blood pressure parameters in SHRs with EDMBPL administration; no significant difference was observed compared to the control. (**b**) Diastolic blood pressure parameters in SHRs with EDMBPL administration; no significant difference was observed compared to the control. (**c**) The determination of the heart rate in SHRs with the administration of EDMBPL; no significant difference was obtained compared with the control. Statistics between the treatments and control were tested by two-way ANOVA, followed by a Tukey test. *n* = 6 animals.

Therefore, EDMB*Pl* was used to evaluate its antihypertensive effect in spontaneously hypertensive rats (SHRs). EDMB*Pl* did not exhibit a significant antihypertensive effect at a dose of 100 mg/kg on the evaluated parameters (Figure 8), i.e., systolic blood pressure (SBP), diastolic blood pressure DBP, and heart rate (HR). This lack of effect may be attributed to factors such as the absorption, distribution, and metabolism of the extract in vivo, which may differ from that observed in an ex vivo model.

### 2.6. Toxicity Assays

#### 2.6.1. Acute Toxicity of EDMBPl

We determined safety with a study of acute and subacute toxicity. When talking about natural products, it is common for people to associate them with safety; however, they must be tested alone or in combination with other pharmaceutical agents [30,31]. In this sense, to demonstrate the safety of EDMB*Pl,* different doses were administered. After 24 h of administration, no deaths were observed in any group. However, at the dose of 5 mg/kg, an abscess in the left arm was observed in one mouse. Due to this and following the OECD 423 guide [32], an average dose of 25 mg/kg was evaluated, with no physical changes or deaths observed in the mice. These results suggest that the abscess observed in the animal were not caused by the extract but rather were likely due to the handling or manipulation of the animals. According to the Globally Harmonized System (GHS), the dichloromethane/methanol extract of *Prosthechea livida* could be tentatively classified into Category 4, suggesting non-toxicity under certain conditions.

#### 2.6.2. Subacute Toxicity of EDMBl

**Figure 9:**
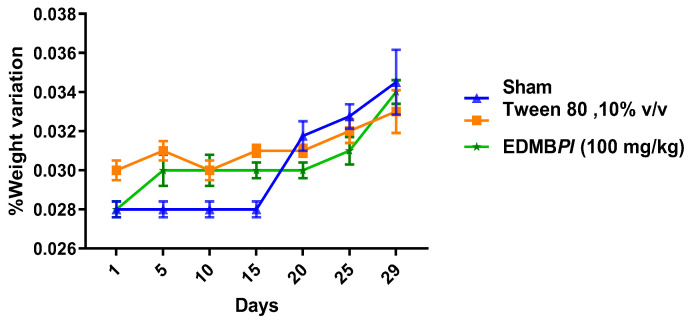
Variation in mouse weights over 28 days of the treatments used (sham, vehicle, and extract). Each bar represents the standard error of the mean, *n* = 5 animals. Statistic differences between the treatments were tested by two-way ANOVA, followed by a Tukey test.

**Figure 10:**
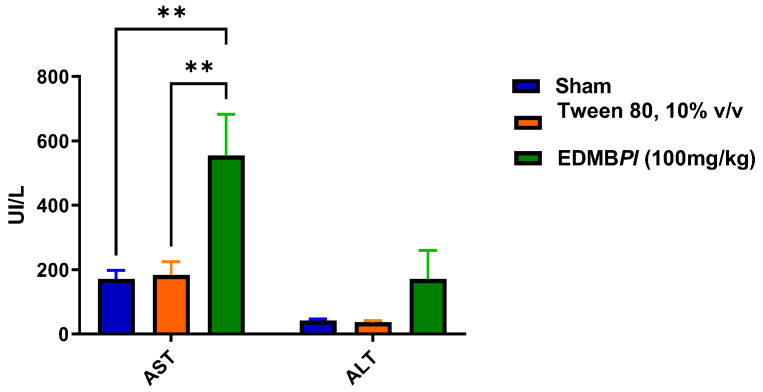
Transaminases (AST, ALT) activity to determine possible liver damage. Each bar represents the standard error of the mean, *n* = 5 animals. Statistically significant differences between the treatments were tested by two-way ANOVA, followed by a Tukey *t*-test. ** *p* = 0.002, 0.003.

**Figure 11:**
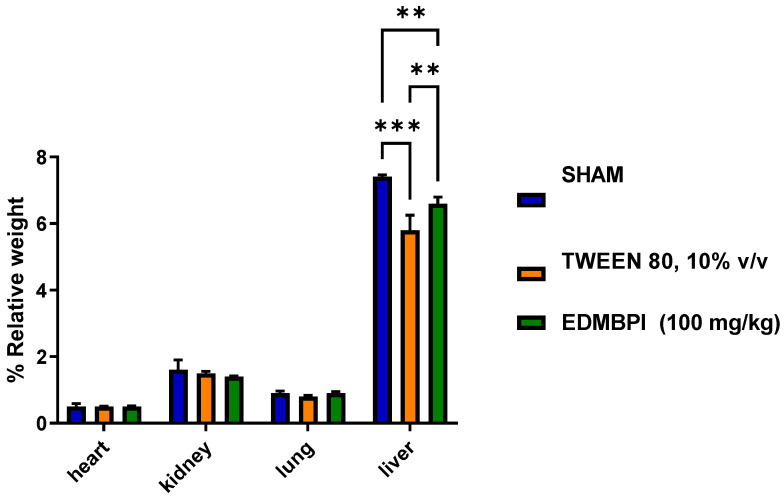
Weight variation in the different organs isolated from mice treated with three different sham (water) treatments, 10% tween 80, and EDMB*Pl*. Each bar represents the standard error of the mean, *n* = 5 animals. Statistically significant differences between the treatments were tested by two-way ANOVA, followed by a Tukey test. ** *p* = 0.008, 0. 005; *** *p* < 0.001.

**Figure 12:**
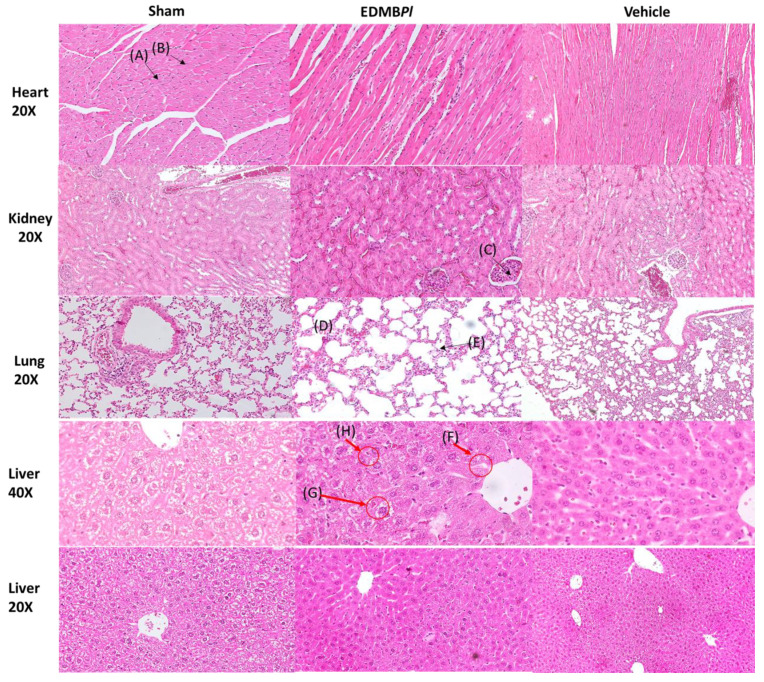
Histological sections of the different organs: the isolated heart, with muscle fibers shown (A), nuclei (B), the kidney glomerulus (C), the lung alveoli (D), and the alveolar septa (E); inflammation is observed in the hepatic artery, as well as the loss of lymphocytes close to the portal vein (F), binuclear cells (G) due to active regeneration by cell replication, hepatocytes with large nuclei (H), and active necrosis minimal, for EDMB*Pl*.

Repeated oral administration of EDMB*Pl* (100 mg/kg/day) for 28 days did not produce characteristic signs of toxicity in either the treated or vehicle groups. The results showed that EDMB*Pl* did not cause changes in body weight, variations in organ weights, or alterations in transaminases levels (Figure 9, Figure 10 and Figure 11) [33]. Histological sections of various organs (heart, lung, kidney, and liver) were examined to identify any cellular damage using hematoxylin and eosin staining. As shown in Figure 12, the heart displayed no damage in the muscle fibers (A) or nuclei (B) across all three treatment groups. Similarly, the kidney showed no damage; the glomeruli (C), responsible for blood filtration and urine formation, and the proximal tubules, involved in solute reabsorption and secretion, appeared normal in all treatments. The lung also exhibited no damage to the alveoli (D) or interalveolar septa (E), crucial for oxygen exchange with the blood. The liver, a key organ in metabolism, required detailed analysis due to changes in transaminase activity. The micrographs in Figure 12 reveal mild inflammation in the hepatic artery, a loss of lymphocytes near the portal vein (F), the presence of binuclear cells (G) indicating the active regeneration of hepatocytes with large nuclei (H), and minimal active necrosis. *Prosthechea livida* has no prior record of toxicity studies; however, this study found no mortality at different doses. The subacute toxicity evaluation indicated no physical or behavioral changes, and the treated animals maintained a stable weight throughout the 28 days of EDMB*Pl* extract administration. Despite this, there was an increase in transaminase activity (Figure 10) and a significant rise in liver weight (Figure 11). Histopathological analysis revealed minimal hepatocellular lesions and signs of active regeneration of hepatocytes. Therefore, it can be concluded that a 28-day treatment with the EDMB*Pl* extract causes minimal liver damage.

## 3. Materials and Methods

### 3.1. Reagents and Drugs

Glucose (G7021), Sucrose (S9378), Nicotinamide (N0636), Streptozotocin (S0130) (STZ), Acarbose (A8980), Glibenclamide, dimethylsulphoxide (DMSO), Noradrenaline hydrochloride (NA), Nifedipine, and Carbamoylcholine chloride were acquired from Sigma-Aldrich Co. (St. Louis, MO, USA). All reagents were prepared before use.

### 3.2. Plant Material

Specimens of *Prosthechea livida* were collected in August 2019 by MBT. Enciso-Diaz in the mountains Otontepec of Ignacio de la Llave in Veracruz State, (21°24′13″ N 98°29′11″ O/21.403611111111, −98.486388888889). A voucher specimen (OE-009) was identified and deposited in the herbarium of the Veracruzana University. Only the pseudobulbs of these specimens were used, which were dry and ground.

### 3.3. Plant Material and Preparation of EDMBPl

Maceration extraction was developed with 81 g dry material and ground in a mechanical grinder; the solvents employed were a mixture of dichloromethane and methanol (50% *v*/*v*), each for 72 h (three times). Later, the macerated was filtered, and a liquid extract was obtained. The solvent was then removed using a rotary evaporator until a dry extract was obtained (R-200, Buchi Labortechnik AG, Flawil, Switzerland).

### 3.4. Pharmacology Assays

#### 3.4.1. Animals

Male ICR mice (25–30 g) were used, organized into groups of five or six animals for antidiabetic and toxicological experiments. The vasorelaxant effect was evaluated using male Wistar rats weighing between 250 and 300 g. To assess the antihypertensive activity, spontaneously hypertensive rats (SHRs) weighing between 300 and 350 g were used, following the provisions of the Official Mexican Standard NOM-062-ZOO-1999 and protocol number B11-20, approved by the Ismael Cosío Villegas National Institute of Respiratory Diseases Ethics Committee.

#### 3.4.2. Oral Glucose or Sucrose Tolerance Tests

A population of 18 mice was used, which were fasted for 8 h with free access to water. Subsequently, 3 experimental groups were formed (*n* = 6 each). The initial blood glucose (T0) was measured with a glucometer (Accu-Chek guide, Roche, Basel, Switzerland) for each group, the positive control group (glibenclamide 5 mg/kg or acarbose 3 mg/kg), the EDMB*Pl* group (100 mg/kg), and the vehicle group (Tween 80, 10% *v*/*v*), 30 min after oral glucose or sucrose (2 g/kg) was administered. Additionally, blood samples were collected from the caudal vein 0, 0.5, 1, 1.5, 2, and 3 h after the administration of the vehicle, positive control, and extract. The percentage variation in glycemia for each group was calculated concerning the initial (0 h) value, according to the formula: %Variation of glycemia = [(Gx − G0)/G0] × 100, where G0 represented the initial glycemia values, and Gx represented the glycemia values at each time, as described [34].

#### 3.4.3. Acute Antidiabetic Effect of EDMBPl

##### Experimental Diabetic Model 

The animals were fasted for 8 h before starting the administration, and subsequently nicotinamide (40 mg/kg) was administered intraperitoneally. After 15 min, streptozotocin (100 mg/kg) dissolved in citrate buffer at pH 4.5 was administered through the same via. After two weeks, hyperglycemia was established by higher blood glucose, which was monitored by a commercial glucometer (Accu-Check guide^®^, Basel, Switzerland). The animals selected for the study were those with blood glucose above 140 mg/dL. Animals were divided into three groups: the vehicle group, orally administered with Tween 80 at 10%; the positive control glibenclamide group (5 mg/kg); and the third group, which received EDMB*Pl* (100 mg/kg). Blood samples were collected from the caudal vein 0 (fasting blood glucose), 1, 3, 5, and 7 h after administration. The percentage variation in glycemia was determined concerning the blood glucose at 0 h, according to the equation mentioned above, with some modifications [34].

#### 3.4.4. Preparation of Aortic Rings

Aortic rings (3–5 mm) were obtained free of connective tissue and fat (in some rings, the endothelium was removed) and suspended by platinum hooks under an optimal tension of 3 g in a Krebs solution (composition was mM: NaCl, 118; KCl, 4.7; CaCl_2_, 2.5; MgSO_4_, 1.2; KH_2_PO_4_, 1.2; NaHCO_3_, 25.0; EDTA, 0.026; glucose, 11.1, pH 7.4), maintained at 37 °C in at atmosphere of 95% O_2_ and 5% CO_2_. Isometric tension was measured and recorded using Grass-FT03 force transducers (Astromed^®^, West Warwick, RI, USA) connected to an MP100 analyzer (Biopac^®^ Instruments, Santa Barbara, CA, USA) (Vergara-Galicia, et al. [25]).

#### 3.4.5. Vasorelaxant Effect of EDMBPl

The aortic rings with and without endothelium were pre-contracted with NA (0.1 μM) for 15 min, then EDMB*Pl* was cumulatively added in different concentrations with 1000 μg/mL every 3 min. Finally, the contractile effect induced by NA and the vasorelaxant effect of EDMB*Pl* were compared with the control groups (nifedipine and carbachol) [29].

#### 3.4.6. Determination of the Mechanism of EDMBPl

For this purpose, three sets of experiments were designed [26].

##### Participation of α-Adrenergic Receptors

Endothelium-denuded aortic rings were incubated with 502 μg/mL of EDMB*Pl* for 15 min, and then NA was cumulatively added at different concentrations (0.1 nM to 1 μM) Finally, the contractile effect induced by NA was compared in the absence (control group) and presence of the extract.

##### Effect of EDMBPl on Blockade of Ca^2+^ Channels

Endothelium-denuded aortic rings were isolated from rats and washed with Krebs solution with KCl (80 mM). Once a plateau was attained, the CRC of EDMBPl-induced relaxation was obtained by adding cumulative concentrations of the extract to the bath (502 μg/mL).

##### Effect of EDMBPl on Extracellular Ca^2+^-Induced Contraction Activated by KCl

To determine whether the inhibition of extracellular Ca^2+ ^influx was involved in EDMB*Pl*-induced relaxation, experiments were carried out in a Ca^2+^-free Krebs solution. Endothelium-denuded aortic rings were washed with Ca^2+^-free solution (30 min) and then rinsed with Ca^2+^-free solution containing KCl (80 mM). The cumulative CRCs for CaCl_2_ (80 μM to 27 mM) were obtained in the absence of EDMB*Pl* (control group) or after 30 min incubation with the test sample (502 μg/mL). Finally, the contractile effect induced by CaCl_2_ was compared to the absence (control group) and presence of EDMB*Pl*.

### 3.5. In Vivo Experiments

#### Antihypertensive Study

An antihypertensive activity study of EDMB*Pl* was conducted in SHRs. Animals were allotted into three groups (six animals each): control rats (CR, group 1), positive control (amlodipine 5 mg/kg, group 2), and treated rats (EDMB*Pl* 100 mg/kg*,* group 3). The treated group received a single intragastric dose of EDMB*Pl* (100 mg/kg). Measurements [blood pressure and heart rate (HR)] were recorded before and after the treatment of the test compound at 0, 1, 3, 5, and 7 h using a tail cuff method (LE 5001 automatic blood pressure meter, (PanLab™ Harvard Apparatus, Barcelona, Spain)). The percentage decrease in HR, SBP, and DBP was calculated.

### 3.6. Toxicity Assays

#### 3.6.1. Acute Toxicity of EDMBPl

Male mice of the ICR strain were used; they were divided into 5 groups of 5 animals with test doses (5, 25, 50, 300, and 2000 mg/kg) of EDMB*Pl*. The animals were fasted for 4 h before the test. The animals were observed individually after administration for the first 30 min and during the following 24 h. They were then observed daily for 14 days. At the end of this period, the number of deaths was determined as % mortality. Finally, according to the results of the extract, it was categorized as established by the Global Harmonization System (GHS) guide [35].

#### 3.6.2. Subacute Toxicity of EDMBPl

Male mice of the ICR strain were used and divided into 3 groups of 5 animals, a control group (tween 80 at 10%), a sham group, and the test dose (100 mg/kg), using repeated doses each 24 h for 28 days, weighting each group, and at 29 days, when euthanasia was performed [36]. The main organs were isolated (heart, liver, lung, and kidney) from each mouse from the experimental and control groups and weighted for the calculation of the relative organ weight, calculated as (% relative weight = organ weight/body weight × 100%). The blood from the heart was obtained by cardiac puncture and processed to obtain plasma, and the activity of alanine transaminase (ALT) and aspartate transaminase (AST) were determined.

#### 3.6.3. Histology

For the staining of the histological sections of the isolated organs, tissue preparation was carried out: the tissues were placed in formalin, and later they were washed and dehydrated in a series of alcohol concentrations, then they were placed in xylol to remove excess alcohol and later placed in molten paraffin, which, once dry, was cut into blocks. The sections were made with a microtome with a thickness of 4 μm and were placed on a slide, passed again through xylol and a series of alcohols to pass through the hematoxylin and eosin stain, washed, and placed in a fixative and a coverslip. Hematoxylin and eosin allowed us to see the nuclei of the cells to determine whether any damage could be observed. Histological slides were examined with a microscope (Zeiss 40×, Oberkochen, Germany).

### 3.7. Statistics

The results are presented as mean ± standard error of the mean (SEM). Data was analyzed by two-way analysis of variance (ANOVA) followed by Tukey’s test. Statistical analyses were performed with Graphpad-prism 2018 software (https://www.graphpad.com/scientific-software/prism/, accessed on 22 April 2024). A statistical difference was considered significant when *p* < 0.05.

## 4. Conclusions

The use of NPs for diabetes and hypertension management remains a promising area of research. Our findings indicate that *Prosthechea livida (Lindl.) W.E.Higgins*, an orchid species with no previously reported studies, holds significant potential as a source of bioactive metabolites for managing these conditions. EDMB*Pl* demonstrated a regulatory effect on glycemia and exhibited a favorable pharmacological safety profile. Additionally, EDMB*Pl* induced vasorelaxation in rat aortic rings via an endothelium-independent mechanism, possibly through the blockade of Ca^2^⁺ channels. These results represent the first evidence that *Prosthechea livida (Lindl.) W.E.Higgins* can promote glucose uptake and improve blood pressure in ex vivo models. Given its dual action on glycemia and vascular tone, EDMB*Pl* may serve as a promising candidate in the development of new therapeutic agents targeting metabolic and cardiovascular disorders. Future studies will aim to elucidate its precise molecular mechanisms, assess its in vivo efficacy, evaluate its potential in preclinical models of diabetes and hypertension, and carry out phytochemical analyses to identify the compounds responsible for these effects.

## Data Availability

Data are contained within the article.

## Abbreviations

LTB4	Leucotriene B4
NP	Natural product
T2D	Type 2 diabetes
STZ	Streptozotocin
DMSO	Dimethyl sulfoxide
EDMB*PL*	Dichloromethane-methanol extract from *Prosthechea livida* bulb
NA	Noradrenaline
EC_50_	Effective concentration 50
NIDD	Non-insulin-dependent
I.P	Intraperitoneal
CRCs	Concentration–response curves
HR	Heart rate
SBP	Systolic blood pressure
DBP	Diastolic blood pressure
GHS	Globally harmonized system
Emax	Maximum effect
GLUT 2	Glucose transporter type 2
OECD	The Organization for Economic Cooperation and Development
ALT	Alanine aminotransferase
AST	Aspartate aminotransferase
OGTT	Oral glucose tolerance test
BKca	Large-conductance calcium-activated potassium
Cav	Voltage-dependent calcium channel
CaCl_2_	Calcium chloride
KCl	Potassium chloride
NaCl	Sodium chloride
MgSO_4_	Magnesium sulfate
KH_2_PO_4_	Monopotassium phosphate
NaHCO_3_	Sodium bicarbonate
EDTA	Ethylenediaminetetraacetic acid
